# Use of weighted multivariate estimates in trials of multi-serotype vaccines to simplify interpretation of treatment differences

**DOI:** 10.1371/journal.pone.0196200

**Published:** 2018-04-27

**Authors:** Merryn Voysey, Andrew J. Pollard, Rafael Perera, Shrijana Shrestha, Stephen Thorson, Thomas R. Fanshawe

**Affiliations:** 1 Nuffield Department of Primary Care Health Sciences, University of Oxford, Oxford, United Kingdom; 2 Oxford Vaccine Group, Department of Paediatrics, University of Oxford, Oxford, United Kingdom; 3 National Institute for Health Research Oxford Biomedical Research Centre, Oxford, United Kingdom; 4 Paediatric Research Unit, Patan Academy of Health Sciences, Kathmandu, Nepal; Sanaria,. Inc, UNITED STATES

## Abstract

**Background:**

Many vaccines contain multiple components. Licensed pneumococcal conjugate vaccines (PCV) contain polysaccharides from 7, 10, or 13 different serotypes of *Streptococcus pneumoniae*. The main outcomes in randomised trials of pneumococcal vaccines are serotype-specific antibody measures. Comparisons are made between groups for each serotype, resulting in multiple separate comparisons of treatment effects which can be complicated to interpret. We investigated methods for computing the overall difference between vaccine groups across all serotypes.

**Methods:**

Pneumococcal antibody concentrations were obtained from a randomised controlled trial of ten-valent pneumococcal vaccine, conducted in Kathmandu, Nepal. Infants received either 2 priming doses of vaccine at 6 and 14 weeks of age followed by a booster (2+1), or 3 priming doses at 6, 10, and 14 weeks of age with no booster (3+0). The overall difference between vaccine schedules across all serotypes was computed at each visit using a multivariate linear model with equal weights for each serotype. Alternative weights were derived from invasive pneumococcal disease cases in Nepal, Bangladesh and Pakistan, and from estimates of the relative invasiveness of each serotype and used in sensitivity analyses.

**Results:**

When 10 separate estimates of treatment differences were computed the ratio of antibody responses for each serotype in the 2+1 group compared with the 3+0 group at 10 months of age varied greatly, with serotype-specific GMRs ranging from 2.80 for serotype 14, to 9.14 for serotype 18C. Using equal weights for each serotype, the overall geometric mean ratio (GMR) was 5.02 (95% CI 4.06−6.22) at 10 months of age, and 1.46 (95% CI 1.14−1.88) at 3 years of age. Using weights based on disease incidence gave GMRs ranging from 5.15 to 6.63 at 10 months of age, and 1.47 to 1.78 at 3 years of age. Using weights based on relative invasiveness gave estimates of 6.81 and 1.59, at 10 months and 3 years respectively.

**Conclusion:**

PCV clinical trial data have a multivariate structure with correlated outcomes for different serotypes. When analysing each serotype separately, the multiple estimates of the treatment effect can complicate the interpretation of trial results. Reporting a single overall estimate which accounts for the correlation between outcomes can simplify such interpretation. Treatment effects can be weighted equally or alternative weights derived from independent data can be used.

Many modern vaccines have multiple components, such as quadrivalent meningococcal group ACWY vaccine or four-component group B meningococcal vaccine, thus these methods are widely applicable.

## Background

Licensed pneumococcal conjugate vaccines (PCV) contain polysaccharides from 7, 10, or 13 different serotypes of *Streptococcus pneumoniae*. Randomised controlled trials of pneumococcal vaccines in infants typically allocate participants to receive multiple priming doses of vaccine with or without a booster vaccination at a later time point. The main outcomes of such trials are serotype-specific antibody measures. Comparisons are made between randomised groups for each serotype, resulting in multiple separate comparisons at each study time point.

Many modern vaccines have multiple components. Acellular pertussis vaccines contain 3 or 5 protein components, meningococcal conjugate vaccines contain up to 4 different meningococcal capsular groups (groups A, C, W, and Y), and pneumococcal vaccines contain up to 23 different serotypes. Trials of multi-component or multi-serotype vaccines result in data which have an underlying multivariate structure. Instead of one outcome per participant (univariate data) there are multiple measures made at the same time on a single blood sample and these measures are correlated within each participant.

The overall interpretation of trial results when there are multiple estimates of a treatment effect is complicated. Antibody measures for some serotypes may be significantly different between randomised groups, and others not. Alternatively, results can go in opposite directions thus there may be significantly higher antibody for some serotypes, but significantly lower antibody for other serotypes.[1]

As early as 2003 it was noted that the multiplicity of endpoints in future PCV trials would complicate statistical analyses.[2] The use of weighted averages was proposed at the time, with different weights suggested. To our knowledge, such methods have not been implemented in immunogenicity trials to date, but are inherently incorporated into estimates from vaccine efficacy (VE) trials. Estimates of VE which are pooled across the serotypes contained in the vaccine are in effect weighted averages, with weights based on disease rates. The vaccine efficacies for serotypes which are responsible for the most disease have the largest impact on the overall estimate in such pooled analyses.[3] Serotypes of pneumococcus are not associated with equal rates of disease in all countries, and serotype-specific disease rates vary from country to country and over time.[4]

We explored the benefits of using multivariate methods to calculate a weighted average−a single overall treatment effect rather than multiple serotype-specific treatment effects. We compared the use of different weights to compute overall estimates based on serotype-specific disease incidence in different geographic contexts and serotype-specific attack rates. Although illustrated here in the context of randomised group comparisons, such overall weighted averages could be applied when comparing other factors such as the weighted average response in males compared with females, or average responses in individuals who are seropositive compared with seronegative at baseline.

## Methods

Pneumococcal serotype-specific antibody concentrations were obtained from an open-label, randomised controlled trial conducted in Kathmandu, Nepal. Ethics approval for the original trial was obtained from Oxford Tropical Research Ethics Committee (OXTREC 61–09 and 1019–13), Nepal Health Research Ethics Committee (NHRC reference number 807 and registration number 28/2013), Nepal Department for Child Health and Immunisations, and the Nepal Department of Drug Administration. Details of the trial are previously published.[5] Briefly, 390 healthy infants aged 40–60 days were randomised to receive the 10-serotype pneumococcal vaccine (PCV10; Synflorix, GSK) in addition to routine immunisations, as either a two-dose prime plus boost (2+1) schedule at 6, 14 weeks, and 9 months of age, or a three-dose prime only schedule at 6, 10, and 14 weeks of age (3+0). Serotype specific antibody levels were measured at 18 weeks (post-priming), 10 months (post-boost), and at 3 years of age (persistence). (Trial registration ISRCTN56766232).

In order to compute overall treatment effect estimates which reflect different geographic contexts we collated data on serotype-specific invasive pneumococcal disease (IPD) cases from Bangladesh, Nepal, and Pakistan, and used these to construct weights.

Weights representing relative disease burden for each serotype were calculated as the proportion of vaccine serotype-specific invasive pneumococcal disease which was attributed to each of the 10 serotypes included in the vaccine. Only PCV10-serotype disease rates were considered as that was the vaccine used in the study.

Estimates of invasive pneumococcal disease rates for Nepal were obtained from surveillance studies prior to the introduction of the vaccine, in Patan Hospital and Kanti Children’s Hospital in Kathmandu, from published[6, 7] and unpublished data. These data were pooled to increase sample size and reduce the number of serotypes with no recorded cases. Estimates of invasive disease rates from unvaccinated populations in Bangladesh and Pakistan were obtained from published papers of hospital based surveillance studies conducted prior to the introduction of the vaccine.[8, 9]

Alternative weights based on attack rates from UK data, were derived from a study of the relative invasiveness of each serotype.[10] The incidence of invasive pneumococcal disease and the incidence of pneumococcal acquisition (nasopharyngeal carriage) were used to construct odds ratios representing the relative invasiveness of each serotype. For serotypes 1 and 5 which were not detected in nasopharyngeal swabs, an incidence of 0.1 per 100,000 child year was substituted in calculations.

### Statistical models

Data were analysed using separate multivariate linear models. The multivariate model is a generalisation of the classical regression model in which a single response is regressed on one or more predictors, to the model whereby multiple response variables are regressed on the predictors.

The generic form of the multivariate linear regression model is denoted as;

Y=XB+E

Where **Y** is a (*n* x *m*) matrix of observations from *n* individuals on *m* response variables; **X** is a (*n* x *k* +1) matrix with columns for k + 1 predictors (*k* regressors plus one intercept); **B** is a (*k* +1 x *m*) matrix of coefficients, and **E** is a multivariate normal matrix of errors such that rows of **E** are mutually independent and identically distributed, each following a zero-mean multivariate Normal distribution with, in general, a non-diagonal covariance matrix. Procedures for statistical inference in multivariate linear models take into account the correlated nature of the outcomes.[11, 12]

We used the above model in which the *m* = 10 response variables in the **Y** matrix are the 10 measures of serotype-specific anti-pneumococcal antibody per participant. One regressor (randomised treatment group) was included in the model, so the **X** matrix has *n* x 2 dimensions (one intercept and one treatment group variable). The vector **b** of *m* = 10 estimated coefficients (from the relevant row of **B**), corresponding to the effect of treatment group on each of the ten serotypes, was weighted according to the vector **w** of proportions of vaccine serotype invasive disease cases which were attributable to each serotype.

The overall weighted mean treatment difference was estimated as **wꞋb,** and its variance as **wꞋVw,** where **V** is the estimated covariance matrix of **E** obtained from the multivariate model.

In the above analysis, **w** was initially treated as fixed, whereas in practice it is estimated from independent data. To investigate the effect of variability in estimated disease rates on the weighted overall difference for each country, we constructed vectors of bootstrapped weights (**w**_boot_) by sampling at random from the empirical distribution of serotype prevalences from that country and using these to re-estimate **w**, as **w**_**boot**_. 10000 overall weighted mean differences were then computed using the bootstrapped weights **(w**_boot_**Ꞌb)** and the variance was estimated as the variance of the bootstrapped estimates plus the mean of the 10000 variances.

Antibody data were log-transformed prior to analysis to normalised the distribution, and thus results are presented as geometric mean ratios (GMR), computed as the anti-log of the log mean difference. All computations were conducted using R version 3.2.2[13] with code and data supplied in the Supporting information (S1 Text and S1 Data).

## Results

### Distribution of invasive disease

Within South Asia there is substantial variation between countries in the distribution of vaccine serotype specific IPD attributed to each serotype (Table 1). Across Asia it is estimated that serotypes 14, 6B, 23F, 1 and 19F are the five commonest invasive disease-causing pneumococcal serotypes, accounting for 50% of IPD cases in this region.[14]

**Table 1:** South Asian estimates of the proportions of vaccine serotype-specific invasive pneumococcal disease due to PCV10 serotypes in unvaccinated populations.

Serotype/ Serogroup	N. cases IPD Patan Hospital Nepal 2001–14 Age 0-14y	N. cases IPD Kanti Hospital Nepal 2004–07 Age 2m-5y	Proportion of PCV10 IPD cases (95% CI) (W_1_)	N. cases IPD Bangladesh 2007–13 (Saha 2015) Age < 5 yrs	Proportion of PCV10 IPD cases (95% CI) (W_2_)	N. cases IPD Pakistan (Shakoor 2014) Age 0-15y	Proportion of PCV10 IPD cases (W_3_)
**1**	37	8	0.5556	46	0.2335	5	0.0962
**4**	1	0	0.0123	4	0.0203	2	0.0385
**5**	12	3	0.1852	31	0.1574	7	0.1346
**6B**	1	1	0.0247	33	0.1675	2†	0.0385
**7F**	0	2	0.0247	13	0.0660	1	0.0192
**9V**	2	0	0.0247	1	0.0051	3†	0.0577
**14**	5	1	0.0741	31	0.1574	8	0.1538
**18C**	2	0	0.0247	13	0.0660	14†	0.2692
**19F**	2	1	0.0370	10	0.0508	7	0.1346
**23F**	2	1	0.0370	15	0.0761	3	0.0577
**Total**	**64**	**17**	**1.0**	**197**	**1.0**	**52**	**1.0**

† Only overall serogroup determined not serotype

Data for 81 cases of invasive pneumococcal disease caused by PCV10-vaccine serotypes (PCV10-IPD) were available for Nepal. Serotypes 1, 5 and 14 were the most frequent vaccine serotypes to cause disease, causing 84% of PCV10-IPD. In Bangladesh, 197 PCV10-IPD cases were available, in which serotypes 1, 5 and 14 accounted for 55% of cases. In Pakistan, where data were only available for 52 cases, 38% were due to these three main serotypes (Table 1).

### Serotype-specific treatment effects estimates

Treatment group differences for each serotype at 10 months of age were correlated with each other as expected. Correlations were all positive and ranged from 0.22 to 0.72. The most highly correlated serotypes were 1, 5 and 7F with correlation coefficients for all comparisons between these three serotypes being greater than 0.7 (Table 2).

**Table 2:** Correlation coefficients for correlations between treatment differences at 10 months of age (post-booster).

	1	4	5	6B	7F	9V	14	18C	19F	23F
**1**	1.000									
**4**	0.609	1.000								
**5**	0.705	0.648	1.000							
**6B**	0.381	0.395	0.400	1.000						
**7F**	0.721	0.672	0.709	0.361	1.000					
**9V**	0.644	0.587	0.652	0.432	0.688	1.000				
**14**	0.483	0.394	0.444	0.320	0.388	0.392	1.000			
**18C**	0.466	0.529	0.450	0.419	0.548	0.564	0.395	1.000		
**19F**	0.423	0.440	0.414	0.367	0.410	0.410	0.305	0.461	1.000	
**23F**	0.610	0.456	0.498	0.218	0.476	0.533	0.345	0.359	0.257	1.000

Correlations were obtained from the variance-covariance matrix of the multivariate model.

When 10 separate estimates of treatment differences were computed the ratio of antibody responses for each serotype in the 2+1 group compared with the 3+0 group at 10 months of age varied greatly, with serotype-specific GMRs ranging from 2.80 for serotype 14, to 9.14 for serotype 18C, illustrating the complications that can arise in drawing a single conclusion from such trials.

### Overall treatment effects estimates

When summarised as a single overall treatment effect with equal weights for each serotype, the difference between the two vaccine schedules at 10 months of age was a 5-fold higher response in the 2+1 arm (GMR: 5.02, 95% CI: 4.06 to 6.22). This overall effect increased to a 6.6-fold higher response in the 2+1 arm when weights based on Nepali IPD cases were applied, (GMR: 6.63, 95% CI: 5.19 to 8.47). This was higher than the overall weighted estimate calculated using Bangladeshi IPD rates (GMR: 5.15, 95% CI: 4.13 to 6.42), or with weights based on Pakistani IPD cases (GMR: 5.89, 95% CI: 4.74 to 7.30) (Table 3).

**Table 3:** Serotype-specific geometric mean ratios (2+1 schedule relative to 3+0 schedule) with overall estimates derived using different weighting structures.

Serotype		GMR at 10 months of age	95% CI		GMR at 3 years of age	95% CI	
**1**		8.13	6.12	10.81	1.77	1.19	2.62
**4**		4.22	3.13	5.70	1.02	0.72	1.44
**5**		7.45	5.67	9.80	1.38	0.97	1.95
**6B**		3.47	2.50	4.83	1.35	0.79	2.29
**7F**		4.04	3.22	5.05	1.61	1.17	2.21
**9V**		4.70	3.51	6.31	1.11	0.70	1.76
**14**		2.80	1.98	3.97	0.88	0.48	1.64
**18C**		9.14	6.90	12.11	4.60	3.05	6.93
**19F**		7.40	5.63	9.73	1.39	0.71	2.72
**23F**		4.04	2.84	5.75	1.36	0.73	2.52
**Overall mean GMR**							
**Equal weights**	(W_0_)	5.02	4.06	6.22	1.46	1.14	1.88
**Country-specific weighted overall GMR**							
**Nepal**	(W_1_)	6.63	5.19	8.47	1.57	1.18	2.08
**Bangladesh**	(W_2_)	5.15	4.13	6.42	1.47	1.13	1.92
**Pakistan**	(W_3_)	5.89	4.74	7.30	1.78	1.37	2.32
**Country-specific weighted overall GMR with bootstrapped weights (10000 bootstrap samples)**							
**Nepal**	(W_1_)	6.63	5.13	8.56	1.57	1.17	2.10
**Bangladesh**	(W_2_)	5.15	4.09	6.48	1.47	1.12	1.93
**Pakistan**	(W_3_)	5.89	4.60	7.53	1.78	1.30	2.49
**Overall GMR with weights based on odds ratios from Table 4**							
**Invasive potential**	(W_4_)	6.81	5.29	8.78	1.59	1.17	2.16

At 3 years of age serotype-specific GMRs ranged between 0.88 (indicating higher antibody in the 3+0 group) for serotype 14, to 4.60 for serotype 18C (Table 3) and the overall difference between treatment groups was GMR:1.46 95% CI: 1.14 to 1.88, indicating higher antibody responses in the 2+1 group.

Bootstrapped confidence intervals were slightly wider (Table 3).

Overall estimates weighted according to invasive potential of each serotype resulted in GMRs (95% CI) of 6.81 (5.29 to 8.78) and 1.59 (1.17 to 2.16) at 10 months and 3 years respectively (Tables 3 and 4). In contrast, when assuming no correlation between serotypes (an invalid assumption), the overall estimates had smaller variances resulting in narrow confidence intervals of 5.02 (95% CI 4.57 to 5.52) and 1.46 (95% CI 1.26 to 1.71) at 10 months and 3 years of age respectively.

**Table 4:** Weights derived from estimates of the invasive potential of pneumococcal PCV10 serotypes.

Serotype/ Serogroup	Sleeman JID 2006 [10] N IPD cases/ N carriers	Cases per 100,000 child years	Carriers per 100,000 child years	OR	Weight (W_4_)
**1**	33/0	0.74	0.1	3.30	0.649
**4**	24/2	0.54	0.71	0.34	0.067
**5**	7/0	0.16	0.1	0.71	0.140
**6B**	62/90	1.39	32.1	0.02	0.003
**7F**	23/3	0.51	1.07	0.21	0.042
**9V**	46/10	1.03	3.57	0.13	0.025
**14**	235/28	5.27	9.99	0.23	0.045
**18C**	49/14	1.1	4.99	0.10	0.019
**19F**	66/74	1.48	26.38	0.02	0.004
**23F**	54/45	1.21	16.05	0.03	0.006
**Total**	**599/267**				**1.0**

OR calculated as *ad/bc* where *a* = incidence of serotype A cases, *b* = incidence of serotype A carriers, *c* = incidence of non-A cases, *d* = incidence of non-A carriers. For serotypes 1 and 5 which were not detected in nasopharyngeal swabs, a value of 0.1 was substituted for the calculation of odds ratios and weights.

## Discussion

These analyses demonstrate how weighted overall multivariate estimators can be used to analyse pneumococcal vaccine trial data, providing summary estimates that simplify the interpretation of trial results and correctly incorporate the correlation between outcomes into the analysis. At 3 years of age, when the majority of serotype-specific differences were non-significant, the overall estimates calculated using multivariate methods were all highly significant regardless of whether weighted equally or whether unequal weighting structures were applied. This illustrates the way that data from multiple serotypes can be combined and statistical power increased.

At 3 years of age the serotype-specific differences between groups in this study varied. Those who received the 2+1 schedule had significantly higher antibody responses for 3 serotypes but for the majority of serotypes group comparisons were non-significant and one GMR favoured the 3+0 group, but was non-significant. Here we have shown that inconclusive or varied findings such as these can be made clearer when summarised as one overall multivariate estimate of effect which in this case indicated an overall statistically significant benefit for the 2+1 group (GMR 1.46, 1.14 to 1.88). Such multivariate estimators can be interpreted as the average difference in antibody response between randomised groups across all serotypes. When the model was misspecified, and serotypes were assumed to be independent, the confidence interval for this estimate was narrower (1.26 to 1.71). This underestimation of the true variation in the data demonstrates that analyses which do not account for the multivariate data structure result in overly precise findings and potentially incorrect conclusions. Therefore, we recommend that analyses that aim to produce combined effect estimates across serotypes should be based on results obtained from multivariate models.

In situations where treatment differences are significantly higher in one group for all serotypes, such as is seen here at 10 months of age, interpretation of trial results is relatively uncomplicated−the schedule which included the booster dose induced much higher antibody responses at 10 months of age (one month post-booster) than the schedule with no booster. There remains however, a large degree of variability in treatment effect estimates which here ranged from an almost 3-fold difference for serotype 14, up to more than 9-fold difference for serotype 18C. These can be summarised using an overall estimate from a multivariate model. Thus at 10 months of age, antibody responses were on average 5 times higher in the 2+1 group.

### Comparisons between equal weights and serotype-specific weights

Overall estimates such as those above, give equal weighting to each treatment difference and thus the point estimate for the overall effect is simply the average of the ten different treatment effects. Equal weighting of serotype-specific treatment effects implies that all serotypes are equally important. This may be suitable in many situations, however, pneumococcal serotypes differ in their invasive potential, and in both their carriage and disease prevalence, thus it may be desirable to compute an overall estimate which factors in some of this variation. Here we have shown that alternative weighting structures for the calculation of overall multivariate estimates can be used when such variation between serotypes is of importance. In the case of Nepal, more than half of all PCV10-IPD cases were caused by a single serotype (serotype 1), thus it might be desirable to more heavily emphasise serotype 1 when calculating combined estimates. In contrast, almost no cases of serotype 4 disease were seen in Nepal thus the vaccine induced antibody response to this serotype may be of limited importance in such a setting. The distribution of IPD cases obtained from surveillance studies can be used to determine weights which are specific to a particular geographic setting. Overall estimate calculated using such weights are tailored to that setting. We chose to use weights based on invasive disease cases in pre-vaccination settings as that was the setting in which the trial was conducted. Trials conducted where routine vaccination is in place can also use invasive disease rates as weights, however estimates of disease cases from post-vaccine introduction would be more relevant.

In these data, the largest serotype-specific estimates of effect at 10 months of age were observed for serotypes 18C and 1 and the lowest estimates of effect were seen for serotypes 14 and 6B. When weighting according to country-specific IPD rates, weights based on Pakistani IPD cases placed more weight on these serotypes with larger treatment effects, than did Bangladeshi weights, as Pakistan had a higher rate of serotype 18C disease. Thus the overall estimate of vaccine effect for Pakistan was higher than for Bangladesh where more weight was given to serotype 6B and less weight to serotype 18C. The weighted treatment effect for Nepal was the largest as almost all weight went on serotypes 1 and 5−two serotypes with large treatment effects. When disease rates for each serotype align closely with the magnitude of treatment differences in a trial, higher overall weighted estimates of effect will result.

We investigated using alternative data to calculate weights based on attack rates for each serotype. Attack rates are odds ratios which represent the varying capacity of pneumococcal capsular serotypes to cause disease once acquired and are computed as the odds of invasive disease for a particular serotype compared to the odds of nasopharyngeal carriage of the same serotype. Serotypes with high attack rates are preferred targets for vaccination as the incidence of disease can be reduced without a large change to the distribution of carried serotypes. This in turn reduces the potential for serotype-replacement–the process whereby a serotype is removed from circulation by vaccinating, and an alternative serotype fills the ecological niche formed, potentially causing just as much disease as occurred prior to vaccine introduction. Attack rates are therefore plausible candidates for weighting of multivariate estimates. Unfortunately, some serotypes, such as 1 and 5, are very rarely detected using nasopharyngeal swabs[4, 15–17] as they are carried less frequently and for shorter durations [18]. When this happens, no odds ratio can be computed, and thus no weight. We substituted zero values with a value of 0.1 per 100,000 child years, for serotypes where incidence of carriage was too low to be detected. This creates weights which emphasise the serotypes which cause disease but are rarely carried, and the choice of value used as a substitute for 0 substantially affects the size of the weight. Our choice of substituting 0.1 instead of 0, resulted in an odds ratio of 3.3 for serotype 1, and 65% of the weight was then assigned to this serotype. Such considerations make weights based on attack rates problematic.

### Variation in estimates of IPD rates

Serotypes associated with cases of invasive pneumococcal disease are not routinely measured in many low-resource settings and invasive disease is relatively uncommon. A limitation of these analyses is that the distribution of serotypes that cause invasive disease in different settings are estimated based on small numbers of cases. For Nepal, only 81 cases were available from surveillance during a 14 year period and for Pakistan the number of cases available was even lower. The variability which results from estimating weights derived from small sample sizes may impact the accuracy of weighted overall estimates. We have combined data from two studies to construct weights for Nepal which adds a further level of variability. To assess the impact of this variability, bootstrapped weights were applied which produced confidence intervals very similar to the non-bootstrapped confidence intervals. The variability in IPD estimates therefore did not substantially impact overall estimates. In spite of the difficulty which exists in obtaining serotype-specific data on invasive disease rates, such data do exist for many countries.[19, 20] Bootstrapped estimates here show that data from even small numbers of cases are useful in constructing weighted estimates however if suitable data do not exist then equal weights can be applied.

## Conclusions

Pneumococcal vaccine clinical trial data have a multivariate data structure with correlated outcomes for different serotypes and these correlations between outcomes should be taken into account when analysing such data. Consideration needs to be given to the weighting structure to apply to such analyses. Weights can be equally distributed across all serotypes, or alternative weights can be applied if suitable independent data from external sources are available. Weighted averages are not only useful when comparing randomised groups in a clinical trial, but could also be used for calculating other overall estimates; such as the average difference between males and females across all serotypes in a vaccine, or the overall response in seropositive compared with seronegative individuals.

Many modern vaccines have multiple components, such as quadrivalent meningococcal group ACWY vaccine or four-component group B meningococcal vaccine, thus these methods are widely applicable. Such analyses are an efficient use of the data, allow for derivation of context-specific estimates, and make interpretation of trial findings simpler.

## Supporting information

S1 Text R code. (DOCX)

S1 Data Data_appendix. (CSV)

## Abbreviations

PCV: pneumococcal conjugate vaccine
GMR: geometric mean ratio
VE: vaccine efficacy
IPD: invasive pneumococcal disease
