# Supplementary material for: Use of weighted multivariate estimates in trials of multi-serotype vaccines to simplify interpretation of treatment differences
Source: PLoS One. 2018 Apr 27;13(4):e0196200. doi: 10.1371/journal.pone.0196200 (PMC5922548; doi:10.1371/journal.pone.0196200)
Supplement: S1 Text — (DOCX) [file pone.0196200.s001.docx]

## S1 Appendix - R Code

mod1 <- lm(cbind(pn1, pn4, pn5, pn6B, pn7F, pn9V, pn14, pn18C, pn19F, pn23F) ~ factor(m10$grp), data=m10)

mu<-coef(mod1)[2,] # vector of mean treatment differences

vcov<-vcov(mod1) #obtain variance-covariance matrix

#remove variances for intercepts - retain variances and covariances of treatment effects

vcov1<-data.frame(vcov[2*(1:10), 2*(1:10)])

v<-as.matrix(vcov1)

#----------overall estimate using equal weights---------------

eq<-rep(0.1, times=10)

eqwt.mn<-t(eq) %*% mu

#variance of equally weighted mean

eqwt.var<-t(eq) %*% v %*% eq

#weights

#each list of weights sums to 1.0 and is in the same order as the linear model.

wt<-c(0.5556,0.0123,0.1852,0.0247,0.0247,0.0247,0.0741,0.0247,0.0370,0.0370)

#weighted mean

wt.mn<-t(wt) %*% mu

#variance of weighted mean

wt.var<-t(wt) %*% v %*% wt
